# Hearts Not Dead after Circulatory Death

**DOI:** 10.3389/fsurg.2015.00046

**Published:** 2015-09-14

**Authors:** Hendrik T. Tevaearai Stahel, Andreas Zuckermann, Thierry P. Carrel, Sarah L. Longnus

**Affiliations:** ^1^Clinic for Cardiovascular Surgery, Bern University Hospital (Inselspital), University of Bern, Bern, Switzerland; ^2^Department of Cardiac Surgery, Medical University of Vienna, Vienna, Austria

**Keywords:** heart transplantation, DCD heart transplantation, DCD cardiac graft, dconation after circulatory death, marginal heart donors, non-heart beating donors

In recent weeks, two pioneering cardiac surgery teams, Dhital and colleagues in Australia (1) and Large and colleagues in the UK (2), reported on what could rapidly become the best and sole affordable solution to the relentlessly growing number of patients awaiting heart transplantation, i.e., transplantation of grafts obtained from donors after death following circulatory arrest, previously termed “non-heart beating donors.” Both groups presented their slightly differing approaches to organ procurement and evaluation at the 2015 Annual Meeting of The International Society for Heart & Lung Transplantation, confirming several successful short-term outcomes for adult, orthotopic heart transplantation with donation after circulatory death (DCD).

As would be expected, a handful of specialists from around the world enthusiastically recognize a realistic complement to “traditional” cardiac donation after brain death (DBD), whereas others may condemn this approach, considering the ethical, practical, and liability aspects as indomitable. Many remain uncertain, possibly because of the apparent paradox, or at least the uncertainty, surrounding transplantation of hearts obtained from donors after “cardiac death.” The Times recently titled “«dead» hearts successfully transplanted into living patients” (2, 3). When it comes to DCD hearts, it appears inappropriate to speak of donation after cardiac death, and potentially even misleading, given that hearts are able to withstand limited periods of warm ischemia without cardioprotection. Although in some countries determination of death based on cardiocirculatory criteria alone is permitted, in many, a prolonged waiting period (e.g., 10–20 min) between circulatory arrest and organ procurement is required to ensure that permanent neurologic damage has occurred (4, 5). A better terminology (6) would therefore be “DCD.”

One important aspect of these reports lies in the fact that this approach goes against the established dogma that irreversible lesions immediately follow the onset of myocardial ischemia. Indeed, the very first heart transplantations, including those performed by Barnard in 1967, were performed with DCD organs (7). However, with the establishment of brain death criteria in the 1970s, DCD hearts were considered “substandard” and therefore rapidly abandoned. More recently, with revival of interest in DCD donation for improving graft availability notably for kidney, liver, and lung, five categories of DCD patients have been established in the modified Maastricht classification system (8). Distinctions between the different categories are based on the specific circumstances surrounding donor death; for example, dead on arrival at the hospital versus anticipated circulatory arrest following withdrawal of treatment. Furthermore, types of DCD patients have also been classified as controlled or uncontrolled according to whether or not the precise course of circulatory death is known. In theory, all DCD patients are potential heart donors, provided that the inclusion criteria are met and ischemic time is limited; however, it is currently unclear whether certain DCD categories would produce better outcomes. It could be argued, for example, that Maastricht category III donors (anticipated circulatory arrest) would be best suited for cardiac graft donation as conditions are controlled and pre-circulatory arrest interventions to limit heart damage may be possible. Yet these donors may undergo asphyxiation, and are therefore expected to experience pressure and volume overload both in the left and right ventricles, possibly leading to cardiac damage (9). This is in contrast to DCD donors who experience circulatory arrest subsequent to exsanguination; in which no overload-related damage, and thereby improved recovery, is expected (10); however, in this case circulatory arrest cannot be anticipated and pre-ischemic cardioprotective interventions are impossible. In addition to graft quality considerations, legal requirements may influence the use of specific DCD donor categories for heart transplantation.

Importantly, all potential DCD grafts undergo unavoidable warm ischemia before cold preservation solutions can be instilled. Notably, the warm ischemic times reported by Dhital for three of their cases ranged between 22 and 28 min, in agreement with previous pediatric DCD heart transplantations (11) and multiple pre-clinical studies demonstrating myocardial warm ischemic tolerance of almost 30 min. Therefore, a window of opportunity remains to protect, harvest, and evaluate the heart. The real challenge of DCD heart transplantation is thus to take advantage of this limited opportunity to counteract risks related to injury resulting from the inevitable ischemia and subsequent reperfusion.

Now these recent reports of DCD heart transplantation are not simply arbitrary provocations, but rather the result of mature, albeit somewhat differing, strategies. Providing that the warm ischemic time was <30 min, Dhital and colleagues instilled hearts with cardioplegic solution before establishment on an *ex vivo*, portable perfusion apparatus primed with the donor’s own (deleukocyted) blood. In all cases, hearts resumed contractile activity and lactate metabolism was assessed for graft evaluation before the decision to proceed with transplantation was taken. Using a somewhat different approach, Large’s group in the UK returned the heart to a beating state *in situ* to enable functional graft evaluation prior to *ex vivo* perfusion. For DCD hearts, graft evaluation is especially critical since no surgeon would take the risk of transplanting a heart without assessing its suitability. Indeed, as compared to traditional procedures in which graft evaluation and the decision to transplant occur before donor circulatory arrest, heart perfusion in DCD, whether it be regional *in situ* or *ex vivo* perfusion, provides the opportunity for graft evaluation after donor circulatory arrest and in a contracting mode. Also, with continuous perfusion on a portable system, assessment can be prolonged, for example in case of ambiguous results, and the decision to transplant can be delayed.

All the same, precise, evidence-based protocols for every step of the DCD heart transplantation process remain to be established. Indeed, conventional methods used with DBD hearts may not yield optimal outcomes as DCD hearts have undergone a period of ischemia prior to procurement and may thus be less tolerant than DBD hearts to additional exposure to damaging conditions. For example, given that DCD hearts are already arrested at the time of procurement and that strategies effective in limiting ischemic heart damage may only be allowed at reperfusion (procurement) for ethical reasons, conventional cardioplegic solutions may not be optimal for DCD hearts. Indeed, Thatte and colleagues have proposed a new cardioplegic solution developed specifically to limit damage to DCD heart grafts (12). Along similar lines, DCD may not be as tolerant to cold, static storage conditions as DBD hearts, since some degree of ischemic damage has already been initiated. Correspondingly, the two groups that recently reported DCD heart transplantations both employed perfusion storage, which, in addition to reducing ischemia during storage, also permits the monitoring of graft function and metabolism, as mentioned above.

Importantly, it may well be that evaluation reliability will be improved with the addition of other parameters in the future. We, and others, have shown that several relatively easily measurable parameters correlate very well with post-transplantation performance. For example, in pre-clinical *ex vivo* perfusion, Colah and colleagues demonstrated that left ventricular hemodynamics may be of potential value for graft evaluation (13); while our group reported that not only hemodynamics, but also biochemical parameters, such as oxygen consumption or markers of necrosis, provide information for the prediction of subsequent post-transplantation contractile recovery (14, 15).

The DCD approach for cardiac transplantation should be contemplated in light of not only new technological advances, such as *ex vivo* graft perfusion which may contribute to a better recovery, but also cardioprotective pharmacological and molecular strategies. It is true that varying ethical and legal limitations, such as the duration of the no-touch period, among countries are likely to influence the precise clinical strategies chosen (8). Strategies may involve the use of cardioplegic solutions and/or organ perfusion, as well as other post-conditioning therapies, whether biologic (cells, proteins, or genes), chemical (drugs), or physical (hypothermia).

The recent reports from Australia and the UK may one day be seen as critical events, marking fundamental changes in the way we view medicine. By transplanting hearts with potentially higher risks of post-operative dysfunction, both transplantation team and patient are asked to accept possibilities of higher post-operative morbidity and mortality, as well as reduced survival as compared to current standards. In other words, increasing the pool of donors with DCD may imply a “weakening” of reported transplantation results, although this “weakening” effect may already be ongoing, considering the increasing use of marginal donors (16), or the increasing donor age in Europe (17). On the other hand, this view concentrates only on patients who receive heart transplants and, as such, reflects performance of the heart transplantation procedure, but not the overall situation of the real problem, i.e., the entire group of transplantation candidates.

Obviously, many aspects must be thoroughly evaluated before DCD heart transplantation can become a routine procedure. Although these recent reports demonstrate the feasibility of this approach as a concrete and rapidly applicable possibility for helping more patients on the waiting list, it would be wrong to generalize without careful ethical, cultural, legal, and practical considerations. Nevertheless, there is an urgent need to find a solution for the rapidly growing number of patients who will never receive a DBD heart [in 2013, approximately 14.5 and 15.5% cardiac transplantation candidates died in the US and Europe, respectively (18)]. We definitely cannot continue to hope that large donation campaigns or political interventions will substantially influence organ availability. Also, we must accept that development of alternative therapies, such as artificial hearts, drugs, stem cells, or tissue engineering, remain long and cumbersome with no guarantee of success. Conversely, the development of a DCD heart transplantation program seems reasonable. Let us work together to make it happen.

## Conflict of Interest Statement

The authors declare that the research was conducted in the absence of any commercial or financial relationships that could be construed as a potential conflict of interest.
